# Why do we need a shift to the transformative paradigm if we are to decolonise global health?

**DOI:** 10.1186/s41256-025-00443-9

**Published:** 2025-09-25

**Authors:** Chloe Tuck, Laura Gray, Robert Akparibo, Richard Cooper

**Affiliations:** 1 Division of Population Health, School of Medicine and Population Health, University of Sheffield, Sheffield, S1 4DA UK; 2 School of Public Health, University of Ghana, Accra, Ghana; 3 Fred Binka School of Public Health, University of Allied Health Sciences, Ho, Ghana

## Abstract

There is growing recognition of the need for global health and associated research to decolonise. Yet discourse so far has overlooked the role that research paradigms play within this. Left unaddressed, this omission could further engender hierarchical approaches in global health research. The transformative paradigm articulates the relationships between evidence, power and oppression. With this acknowledgement, we can strive for positive social change through research. In this commentary, we argue the importance of considering the transformative paradigm in efforts to decolonise global health research. We provide an initial overview of key terms in this debate, before exploring what is meant by a research paradigm in more detail and then arguing that a transformative paradigm offers unique and powerful opportunities to address enduring colonial inequities in global health research; we then illustrate how this was applied in a recent mixed methods study which explored experiences and barriers to accessing cancer treatment in Ghana. We show how researcher sensitivity to historical injustices and community-based values were vital to our study design and also in specific methods like a participatory creative task and qualitative interviews. This commentary is important as part of the wider debate about decolonising global health and provides a unique critical insight into how research and how particularly research paradigms are of importance in this task, offering suggestions based on a transformative paradigm.

## Background

There is increasing realisation that global health has been established on colonial legacies [1]. Our histories and past events continue to shape our present positions in relation to access to power, knowledge, justice and health [1, 2]. If not acknowledged and addressed, this will only exacerbate inequities further [2]. There have been numerous calls to decolonise the discipline of global health [3]. Decolonisation refers to critically examining how historical, economic and cultural values and structures that shape societies globally are influenced by colonialism, and actively working to counter this [4]. Most notable in the wake of the 2020 pandemic, Abimbola et al. [1] have called for a change in mindset, recognizing colonialism’s role, our position and privileges as researchers. This calls for redressing power by empowering rather than ‘fixing’; the latter implies those who have been marginalised in society are deficient rather than oppressed. There is also a recognition of the impact that the words we use have and in particular the need to move away from ‘savourist’ terminology [1, 5]. Researchers have also questioned epistemic injustice in relation to how evidence is gathered and shared [6]. They highlight concerns in relation to positionality, noting that global health research is often led and promotes values and frameworks created by those in the Global North. These do not reflect community values or prioritise local expertises [6]. The term gaze refers to the dominant audience of the research, which often overlooks the communities which the research attempts to support [5]. Meanwhile, the work of Temple [7] pays attention to the power hierarchies that are associated with language and more specifically translation in research, which can negatively impact on how a participant may be perceived to audiences. Chaudhuri et al. call for a systems overhaul in global health, which is in itself inherinently colonial [8]. They advise this movement to be more introspective and draw on social theory [8]. Whilst not challenging any of these existing critiques of the global health decolonisation agenda, in this paper, we argue that for global health to decolonise it must also embrace new paradigm in research, an aspect that has not been highlighted sufficiently. In this commentary, our aim is to provide an additional insight into current decolonising global health debates by arguing that attention must be given to the research paradigms that are used to understand global health and that using approaches like a transformative paradigm offers important opportunities to address injustice.

## Traditional research paradigms

In research, a paradigm can be seen as a worldview or shared set of beliefs that inform how we can interpret research [9]. This concerns how knowledge is seen to be understood and what is valued [9]. This is central to the methodology and approach that researchers take, and what inferences they can make. This consists of four key dimensions: ontology, epistemology, methodology and axiology [9, 10]. Ontology concerns the nature of reality and what can be known. Epistemology describes how we can aquire knowledge. Methodology encompasses a description of the research design, approach and methods used to collect and interpret data, whereas axiology provides an ethical stance.

Global and public health have evolved as a discipline for the most part from clinical medicine and this is argued to be a fundamental concern given the assumptions that underpin the latter. Clinical medicine has traditionally and arguably continues to apply a paradigm that may be suited to biomedical research on patients, in a medical setting and often under controlled conditions. Commonly, even if not explicit, medical research assumes a positivist paradigm. This assumes an ontology that a world of objects exists to be known and an epistemology that we gain understanding through observation and reason [9]. This does not consider the role of society in shaping how we come to know truth. Leading on from this, positivism seeks beneficence, to maximise good outcomes (under the proviso that there is one good to know). These ethical principles are tailored to what was seen as appropriate within a traditional (hierarchical) doctor-patient relationship, but often reduced to a series of bureaucratic hurdles [11]—from personal experience these can include obtaining a large number of correctly signed administrative forms, printing several hundred page documents in multiple copies, and submission by preset dates with long waiting time.

This is not to say that there are no alternative paradigms that may offer more appropriate and sensitive way of conducting global health research. Examples of these are interpretivism, used commonly in qualitative research and pragmatism, which is suited to mixed methods that bring together different forms of evidence [9]. But these do not inherently address the social injustices in evidence generation and seek to oppose these. Moreover, research needs to start with questions of value. If we are to decolonise, global health research needs to be built on the values of communities rather than colonial legacies. For these reasons there is a need for global health to adopt a paradigm better aligned to the critical approach needed to decolonise the discipline. Here we argue an appropriate starting point can already be found in social justice research and the transformative paradigm [10, 12].

## Transformative paradigm opportunities

In this paradigm, knowledge is embedded in social position and power, and evidence and research are sought to redress this injustice [10, 12]. Ontologically, there is recognition that there may be multiple realities and that these are socially constructed. In this regard, it is similar to the interpretivist paradigm [9]. However, the transformative paradigm goes further in acknowledging underlying social positions that contribute to shaping these realities [10]. There is a need to be explicit about the power and privilege inherent in different forms of reality. Epistemologically, knowledge requires an interactive relationship between the researcher and participant, and this cannot be disentangled from the social and historical context of knowledge [10]. Methods should not only accommodate cultural complexity but also seek to address power imbalances. This can be qualitative or quantitative and includes participatory and creative methods. These move away from the traditional forms of evidence prioritised in colonial discourse, instead accommodating those who have been marginalised. Critically, the transformative paradigm’s axiology is grounded in respect for the values of communities [10]. It considers community values and ethics in promoting social justice [10]. This is crucial for global health research if we are to move away from the epistemic injustice in knowledge domination by the Global North [5]. Cornwall and Jewkes define the difference between traditional and participatory approaches through asking: who (which communities—academic other otherwise) defines the research problems and takes ownership of the results? [13].

## Illustrating alternative approaches

Other disciplines have sought anti-oppressive approaches that are led by community values. One example is indigenous research, where communities such as the San in South Africa and aboriginal groups in Australia have established their own ethics boards [14, 15]. Similar approaches are argued to be needed in global health to redefine ethics and purpose in such research, and in doing so, counter the colonial narratives entrenched in global health and associated research. Further illustrating how this can be applied in the global health setting, we draw on our recent mixed methods study undertaken in Ghana by Tuck and colleagues, which explored the experiences of access to cancer services [16]. We were operating within multiple layers of inequity, which are known to be experienced by underserved patients [17] and extending to many other members of the community including staff supporting the study. Further, we were aware of the history of colonial oppression that continues to influence how the global health sector operate [18]. This influenced how knowledge was both understood and valued and was predicated on an ontological position which recognised different realities that are influenced by positionality and power. In designing the study, awareness of colonial legacies and an English language gaze on evidence [5] led us to regard traditional interviewing techniques as suboptimal, subordinating knowledge that may not be articulated effectively in English. Instead, an epistemology was adopted which acknowledged that ways of knowing are relational and reflect social and historic experiences, including experiences of power. This influenced our choice of methods, specifically participatory methods, that opened up opportunities for diverse forms of evidence, including creative tasks by participants. We also reflected on how different forms of evidence have different levels of influence, which is particularly important for social change. The impact of different forms of evidence is inherently limited by multiple systems of power and dominance in society. While a mixed methods approach was used overall, but being consistent to the importance of a transformative paradigm, the study acknowledged the specific value of using qualitative methods alongside creative tasks. Acknowledging that different realities are influenced by power and oppression led us to reflect on the ethical aspects of social position and our responsibility to represent and acknowledge these appropriately. Thus, it was important that the axiology was embedded in community values. An example of this was how the research sought ways to be reciprocal and bring voice to the community. From the creative task component of the study linked to patient interviews, an art exhibition was created for the participating oncology centre and a booklet produced to communicate patients’ experiences and also influence and inspire others.

Although not possible in this example in Ghana, a transformative paradigm approach typically uses a community-value laden axiology by involving community members in the research process, such as using a community advisory committee or involving mioritised community researchers or those with lived experience of oppression in the data collection and analysis [19]. This example helps illustrate why such a paradigm change is needed. The transformative paradigm allows us to identify injustice related to knowledge and seek to undertake research that addresses such power imbalances. Only by removing this blind spot and acknowledging the positionality of evidence can we appropriately consider how to conduct global health research. Moreover, the transformative paradigm emphasises the importance of social accountability (recognising the researchers role and how they can positively contribute to society through their research). This can ensure accountability to the communities that it seeks to support.

## Conclusions

To overhaul the colonial base of global health—which intersects with other forms of oppression, including not limited to racism, capitalism, ablism and sexism—and to move to giving agency to those who have been marginalised and made vulnerable, the transformative paradigm provides an insightful philosophical underpinning. It acknowledges that social realities are shaped by position and power and that knowledge requires sensitivity to these relationships. It advocates for research to counter social and health injustice which is laden in the values of the communities who have been oppressed and under-represented. Only with such a change in mindset is the potential to transform global health possible, and a paradigm shift is a key way in which this could be achieved. Resistance to such paradigm shifts may also reflect a broader resistance to overhaul colonial dominance in knowledge legitimacy.

## Data Availability

This comment did not involve any dataset.
